# SIV Env RhmAbs + N-803 at ART initiation prolongs viral decay without disrupting reservoir establishment in SIV-infected infant macaques

**DOI:** 10.1371/journal.ppat.1012863

**Published:** 2025-01-10

**Authors:** Omotayo Farinre, Tzoalli Anaya, Alexis C. King, Kedan Endrias, Anne H. Hébert, Alison L. Hill, Sherrie Jean, Jennifer S. Wood, Stephanie Ehnert, Shan Liang, Gregory M. Laird, Rosemarie D. Mason, Mario Roederer, Jeffrey T. Safrit, Maud Mavigner, Ann Chahroudi

**Affiliations:** 1 Department of Pediatrics, Emory University School of Medicine, Atlanta, Georgia, United States of America; 2 Institute for Computational Medicine, Johns Hopkins University, Baltimore, Maryland, United States of America; 3 Emory National Primate Research Center, Emory University, Atlanta, Georgia, United States of America; 4 Department of Pathology and Laboratory Medicine, Emory University School of Medicine, Atlanta, Georgia, United States of America; 5 Accelevir Diagnostics, Baltimore, Maryland, United States of America; 6 Vaccine Research Center, National Institutes of Allergy and Infectious Diseases, Bethesda, Maryland, United States of America; 7 ImmunoTechnology Section, National Institutes of Allergy and Infectious Diseases, Bethesda, Massachusetts, United States of America; 8 ImmunityBio, Culver City, California, United States of America; 9 Center for Childhood Infections and Vaccines of Children’s Healthcare of Atlanta and Emory University, Atlanta, Georgia, United States of America; University of Wisconsin, UNITED STATES OF AMERICA

## Abstract

The latent viral reservoir remains the major barrier to HIV cure, placing the burden of strict adherence to antiretroviral therapy (ART) on people living with HIV to prevent recrudescence of viremia. For infants with perinatally acquired HIV, adherence is anticipated to be a lifelong need. In this study, we tested the hypothesis that administration of ART and viral Envelope-specific rhesus-derived IgG1 monoclonal antibodies (RhmAbs) with or without the IL-15 superagonist N-803 early in infection would limit viral reservoir establishment in SIV-infected infant rhesus macaques. Following initiation of ART at 1–2 weeks after oral SIV_mac251_ infection, we observed biphasic decay of viremia, with first phase decay significantly faster in the ART + SIV RhmAbs-treated group compared to controls that received only ART. In contrast, the addition of N-803 to ART + SIV RhmAbs significantly slowed both the first and second phase viral decay compared to the ART only group. Treatment with a single dose of N-803 resulted in increased frequency of Ki67 expressing NK, CD8+, and CD4+ T cells. Levels of intact SIV proviruses in CD4+ T cells from blood, lymph nodes, and rectum at week 48 of ART did not differ across groups. Similarly, the time to viral rebound following ART interruption was not impacted by the experimental treatments. These results support the concept that the rebound-competent viral reservoir is formed within days after infection and that targeting only productively infected cells for clearance near the time of ART initiation, even during acute infection, may be insufficient to limit reservoir establishment.

## Author summary

A major goal of contemporary HIV research is to identify a treatment approach that reduces or eliminates infected cells from the body. For newborns with perinatal infection, there is a unique opportunity to intervene early in the first days to weeks of life with antiretroviral therapy (ART) combined with potential curative agents. Here we used a well-established infant monkey model of perinatal HIV infection to evaluate the safety and efficacy of two immunotherapies administered with ART. We found that the IL-15 superagonist N-803 prolonged the lifespan of infected cells, but this did not improve their elimination by passively administered antiviral antibodies. These results suggest that strategies to more effectively reactivate latent virus during initial treatment with ART in infants are likely needed to disrupt reservoirs. An HIV cure for children remains elusive; however, our research efforts in relevant monkey models continue to provide insights into approaches that may or may not work in people.

## Introduction

An estimated 120,000 new HIV infections were reported in children between the ages of 0–14 in 2023, with ~50% of vertical transmissions during the breastfeeding period [1]. When available and strictly adhered to, antiretroviral therapy (ART) is remarkably effective at controlling viral replication but is not a cure. Interruption of ART leads to viral resurgence due to reactivation of rebound-competent virus from latently infected cells (“the reservoir”) that are not eliminated by ART or the immune system [2–5]. The challenge of curing HIV is heightened for infants with vertically acquired infection due to the current clinical guidance to treat with ART from birth to death. Infant infection therefore presents a unique opportunity to intervene early towards the goal of a cure as the timing of HIV acquisition is frequently known.

Immunotherapeutics, such as monoclonal antibodies directed against the viral Envelope (Env), can target both circulating virions and cells expressing HIV antigen before viral suppression on ART [6]. As such, treatment with antiviral antibodies at the time of ART initiation may restrict reservoir seeding. Further, intervening early in infection with immunotherapeutics in combination with ART offers the advantage of reduced viral diversity and preservation of antiviral immunity. A study of the HIV Env CD4 binding site (CD4bs) broadly neutralizing antibody (bNAb) 3BNC117 dosed shortly after ART initiation demonstrated faster HIV RNA decay the bNAb treatment groups compared to ART alone but did not find a smaller intact HIV proviral reservoir [7]. Interestingly, however, when analytical treatment interruption (ATI) was performed after a year of ART, one individual in the group that received 3BNC117 and the histone deacetylase (HDAC) inhibitor Romidepsin around the time of ART initiation maintained undetectable plasma viral loads for at least 12 weeks [7].

In infants the main interventions tested to limit reservoir establishment have been early and very early ART initiation [8,9]. A single completed study added the CD4bs bNAb VRC01 to ART in 7–14-week-old infants but did not find differences in HIV DNA copies/million PBMC compared to ART alone after 14 weeks of treatment, possibly due to pre-existing VRC01 resistance [10]. Post-exposure treatment with bNAbs has been studied in a newborn macaque model, with half of macaques given VRC07-523LS + PGT121 (targeting the CD4bs and V3 glycan loop of HIV Env, respectively) 48 hours after high-dose SHIV challenge showing low or no viral DNA in tissues ~6 months later [11]. Collectively, these data indicate that limiting reservoir seeding and/or preventing viral rebound are possible, although the timing of and specific interventions used are certain to be critical to engendering these desired outcomes.

We have previously demonstrated that an SIV Env-specific rhesus monoclonal antibody (RhmAb) cocktail reduced the lymph node replication competent viral reservoir when combined with non-canonical NF-kB activation to reverse latency during ART suppression [12]. These antibodies, with specificity for the V2 glycan loop (ITS09.01), CD4bs (ITS102.01 and ITS103.01), and membrane proximal external region (MPER; ITS113.01) of SIV Env, were isolated from SIV-infected rhesus macaques and expressed as full-length rhesus IgG1 modified to encode the LS-encoding mutation (M428L/N434S) to extend half-life. The CD4bs RhmAbs ITS102.01 and ITS103.01 can be considered bNAbs given that they are capable of neutralizing tier 1–3 SIV strains, while ITS113.01 neutralizes tier 1 and 2 SIV (including SIV_mac251_), and ITS09.01 is only capable of tier 1 neutralization [13,14]. Due to their Fc domain, each of these SIV RhmAbs also has non-neutralizing effector functions, including antibody dependent cellular cytotoxicity (ADCC) and antibody-dependent neutrophil phagocytosis (ADNP), with ITS09.01-LS being the best at ADNP [12–14].

N-803, an IL-15 superagonist, is a known anti-tumor agent that has been shown to stimulate the expansion of NK and CD8+ T cells, as well as promote trafficking of these cells to lymph node B cell follicles [15–20]. Although conflicting evidence exists [20], N-803 has also been suggested to reverse HIV/SIV latency [18,19]. For these reasons, N-803 has been explored for its potential as a therapeutic agent in HIV cure strategies, both during ART-suppressed infection and at ART initiation [16,21,22] (NCT04505501).

Here we tested the hypothesis that early administration of ART in combination with the SIV Env-specific RhmAb cocktail described above plus N-803 would destabilize and reduce reservoir establishment using a previously established model of postnatal SIV transmission in infant rhesus macaques [23–25]. This approach, which we termed “surge and purge”, used N-803 to activate and expand NK and CD8+ T cells and possibly reverse latency in CD4+ T cells (the “surge”) [18–20]. The immune enhancement mediated by N-803 was paired with passive administration of the cocktail of anti-SIV RhmAbs with the goal of eliminating antigen-expressing SIV-infected CD4+ T cells through ADCC/ADNP and other mechanisms (the “purge”). A subset of SIV RhmAbs in the cocktail (ITS102.01-LS, ITS103.01-LS, and ITS113.01-LS) was also expected to neutralize circulating SIV_mac251_ virions. As such, in the setting of acute SIV infection in infants, treatment with N-803 + SIV RhmAbs was predicted to accelerate the loss of infected cells during the decay of viremia that follows ART initiation. In fact, we show that the selected interventions did not limit reservoir seeding nor modulate viral rebound following ART interruption, indicating that alternate approaches to target reservoir formation are needed.

## Results

### SIV infection and immunotherapies at ART initiation

**Fig 1:**
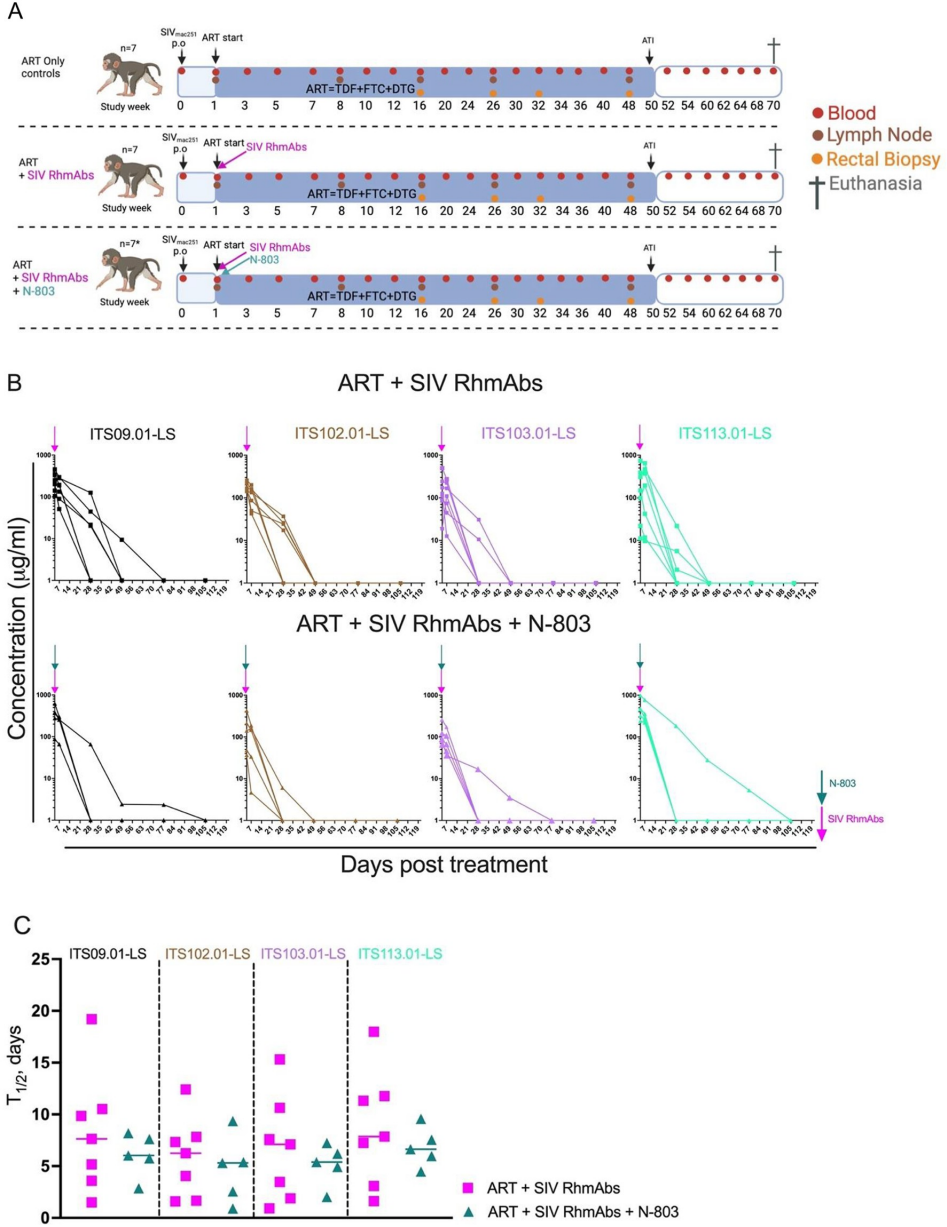
SIV infection and immunotherapies at ART initiation in infant macaques. (**A**), Experimental design. Twenty-one infant rhesus macaques were orally challenged with SIV_mac251_ at 4 weeks of life. ART was started 1–2 weeks post infection. RMs were then divided into three groups: ART only controls (n = 7); ART + SIV RhmAbs (n = 7) and ART + SIV RhmAbs + N-803 (n = 7; *2 animals were prematurely euthanized due to adverse events resulting in a final n = 5). Animals received a single dose of SIV RhmAbs at 20 mg/kg s.c. each and a single dose of N-803 at 0.1 mg/kg. Both SIV RhmAbs and N-803 were administered on the same day as ART was initiated. ATI, Analytical Treatment Interruption. Created with Biorender.com. (**B**), Plasma concentrations of each SIV RhmAbs in μg/ml in the ART + SIV RhmAbs and ART + SIV RhmAbs + N-803 days post treatment. (**C**), Half-life of SIV RhmAbs in ART + SIV RhmAbs and ART + SIV RhmAbs + N-803 treated group. Individual data points are plotted with horizontal line drawn at the median.

Twenty-one four-week-old infant rhesus macaques were orally challenged with SIV_mac251_, and ART was initiated following confirmation of infection between 7–17 days after successful challenge across all groups (Fig 1A). Most infants required 1–2 weekly challenges to become viremic, although a couple of macaques required up to 3 and 4 oral inoculations (S1 Table). ART was maintained until week 50 of infection and then an analytical treatment interruption (ATI) was performed (Fig 1A). At the start of ART, seven infant macaques received a single subcutaneous (s.c.) dose of the IL-15 superagonist N-803 at a dose of 0.1 mg/kg. In addition, a 20 mg/kg s.c. dose of each of the SIV RhmAbs ITS09.01-LS, ITS102.01-LS, ITS103.01-LS and ITS113.01-LS (recognizing V2, CD4bs, CD4bs proximal, and MPER sites respectively on Env) was administered to the 14 infants in the two experimental groups. Five of seven infant macaques treated with ART + SIV RhmAbs + N-803 experienced a range of mild to severe adverse effects (S2 Table). In two cases, the severity of the symptoms necessitated euthanasia. A thorough review by study staff, veterinarians, pathologists, IACUC, and outside experts could not definitively distinguish the specific intervention that led to the adverse events and, in the case of the two euthanized animals, non-study related health issues may have contributed to their rapid decline. Out of an abundance of caution, further doses of N-803 were not administered.

### Plasma concentrations of SIV RhmAbs in infant rhesus macaques

Plasma RhmAb concentrations were measured longitudinally. All SIV RhmAbs peaked at day 4 (first measurement) and declined rapidly thereafter to undetectable levels by week 7 post infusion, except for RNq22 where the RhmAbs persisted up to week 15 in the N-803 treated group (Fig 1B). Median half-life of the SIV RhmAbs ranged from 6.3–7.9 days in the group treated with SIV RhmAbs only and 5.3–6.7 days in the group treated with both SIV RhmAbs + N-803 (Fig 1C and Table 1), without significant differences between the groups. The median maximum concentration (C_max_) for ITS09.01-LS, ITS102.01-LS, ITS103.01-LS and ITS113.01-LS was 202 μg/ml, 156 μg/ml, 126 μg/ml, and 195 μg/ml, respectively, for macaques treated with SIV RhmAbs only. Median C_max_ for each RhmAb was generally similar for animals treated with SIV RhmAbs + N-803 (Table 1).

**Table 1:** Pharmacokinetic parameters of SIV Env RhmAbs.

Groups	ITS09.01-LS		ITS102.01-LS		ITS103.01-LS		ITS113.01-LS	
	C_max_* (μg/ml)	T_1/2_* (days)	C_max_ (μg/ml)	T_1/2_ (days)	C_max_ (μg/ml)	T_1/2_ (days)	C_max_ (μg/ml)	T_1/2_ (days)
ART + SIV RhmAbs	202	7.6	156	6.3	126	7.1	195	7.9
ART + SIV RhmAbs + N-803	368	6.0	159	5.3	90	5.4	456	6.7

*Values represent the median of the parameter measured.

### Plasma viral decay on ART in infant macaques

**Fig 2:**
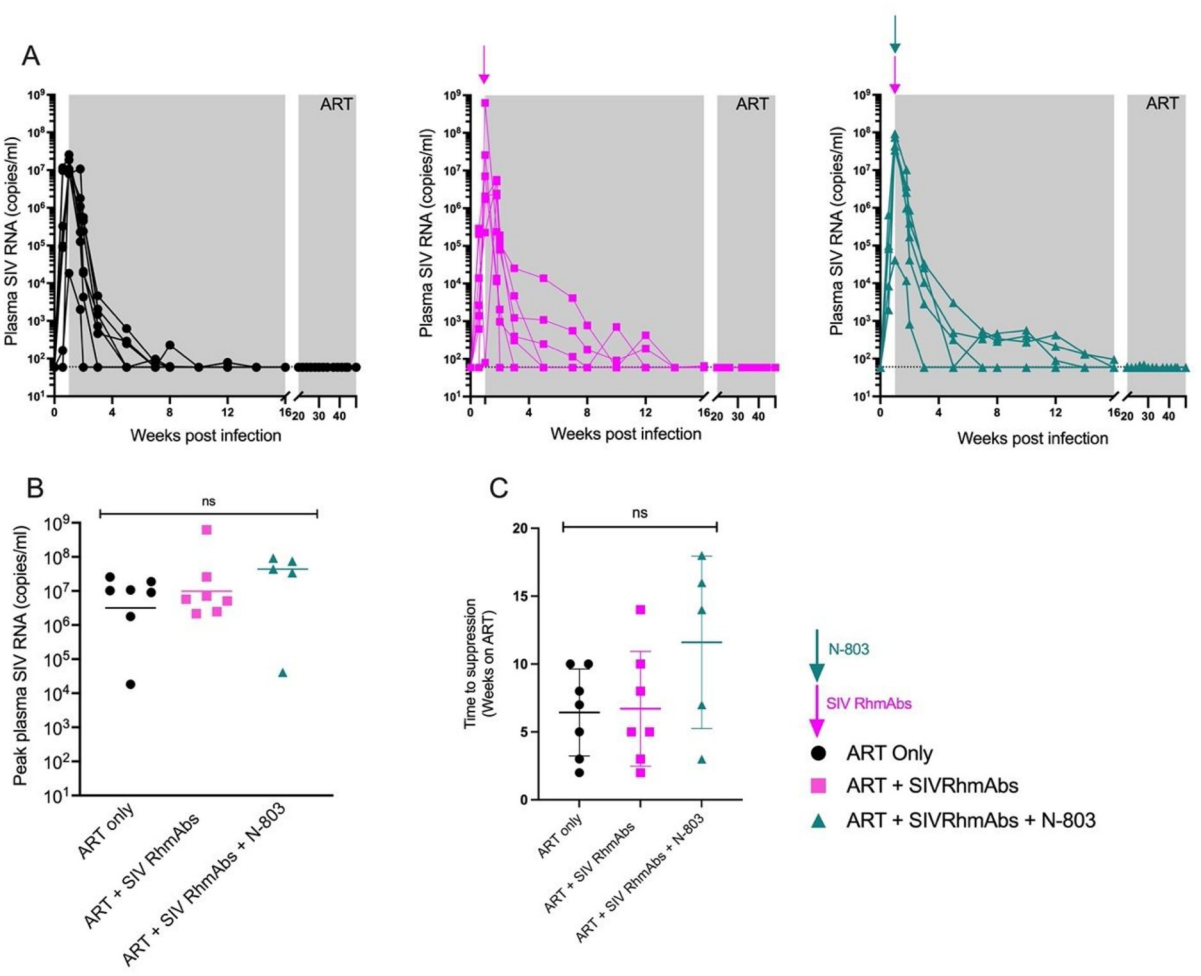
Plasma SIV viral loads before ART were similar across groups. (**A**), SIV RNA copies in plasma were tracked longitudinally following infection with SIV_mac251_ and treatment with ART. Grey shading indicates the period of ART administration. (**B**), Peak viral loads were compared across groups by one-way ANOVA. Individual data points are plotted with horizontal line drawn at the median. (**C**), Time to viral suppression, defined as three consecutive undetectable timepoints, was compared across groups by one-way ANOVA. Individual data points are plotted with mean and SD shown. ns = not significant; p > 0.05.

**Fig 3:**
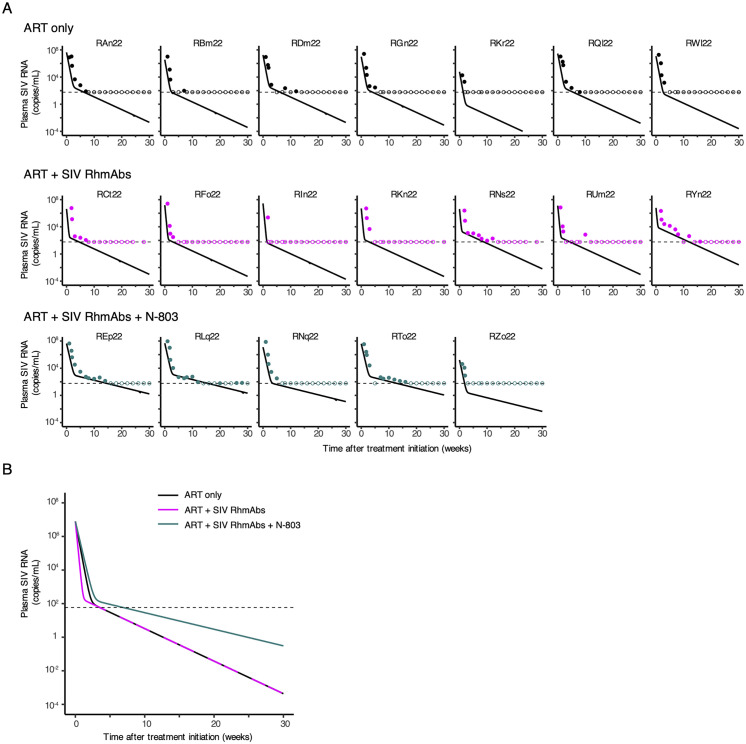
Faster SIV viral load decay in the ART + SIV RhmAbs group and opposite effect with the addition of N-803. (**A**), Viral load values and individual fit trajectories for each animal after ART initiation are shown, with dashed lines indicating the lower limit of detection. (**B**), Simulated viral load decay trajectories by treatment group. Group mean trajectories show differences in the first and second phase decay rates. The trajectories for the ART only control and the ART + SIV RhmAbs treatment groups overlap after the first phase decay since there were no inferred effects of the SIV RhmAbs on the second phase decay rate. Dashed lines were used to indicate the overlap between the ART only group and the ART + SIV RhmAbs group.

Plasma viral load peaked between 10^4^−10^9^ SIV RNA copies per ml pre-ART with no significant differences across groups (Fig 2A and 2B). Following ART initiation, we did not observe significant differences in time to viral suppression across groups (Fig 2C), although 3/4 RMs with the longest time to viral suppression were in the ART + SIV RhmAbs + N-803 group (Fig 2C). We next compared the kinetics of viral decay following ART initiation across the different groups. Plasma SIV RNA values for each animal were fit jointly to bi-exponential decay curves using a mixed-effects framework, where RhmAbs and N-803 were assumed to have independent and additive effects on the parameter values and significant random effects and treatment effects were identified with a combined forward-backward selection. Viral load values and individual fit trajectories for each animal after treatment initiation were plotted to show the inflexion points in the bi-phasic decay of plasma viral loads (Fig 3A). Relative to the ART only group, the best fit model suggested that SIV RhmAbs increased the first phase viral decay by 0.65/day ([0.59, 0.71], p < 0.001; Table 2). In contrast, the addition of N-803 decreased both the first and second phase decay rates (by -0.79/day [-0.85, -0.74], p < 0.001 and -0.031 [-0.050, -0.012], p = 0.001, respectively) (Table 2). The predicted half-life of productively infected cells was thus shorter in the SIV RhmAbs group (0.51 days) and longer in the SIV RhmAbs + N-803 group (1.2 days) versus ART only (0.96 days) during first phase decay. During second phase decay, the half-life of productively infected cells in the SIV RhmAbs + N-803 group was 21 days versus 11 days in the ART only group. The simulated viral load decay across groups shown in Fig 3B highlights these differences and suggests that the opposing effect of N-803 on viral decay during ART is greater than that of the SIV RhmAbs.

**Table 2:** Estimated model parameters for viral decay on ART. Values represent mean and 95% confidence intervals of estimated parameters from a mixed effects biexponential decay model. Relative to the baseline ART only group (first column), treatment effects for both SIV RhmAbs and N-803 were estimated (second and third column), which contribute to the overall parameters for the ART + SIV RhmAbs and ART + SIV RhmAbs + N-803 groups (fourth and fifth columns). For treatment effects, blank cells indicate that no significant effect of the treatment on that parameter was identified. Half-lives were calculated from decay rates as *T*_*1/2*_ = *ln(2)/b*.

		ART	SIV RhmAbs	N-803	ART + SIV RhmAbs	ART + SIV RhmAbs +N-803
		Group Parameter	Treatment effect	Treatment effect	Group Parameter	Group Parameter
**Pre-treatment viral load (log**_**10**_ **copies/mL)**	*log*_*10*_ *(V*_*0*_ *)*	6.9 [6.5, 7.3]	--	--	--	--
**Proportion of infected cells in long-lived state**	*log*_*10*_ *(A)*	-4.4 [-5.0, -3.9]	--	--	--	--
**First phase decay rate (days** ^**-1**^ **)**	*b*_*1*_	0.72 [0.68, 0.77]	0.65 [0.59, 0.71]	-0.79 [-0.85, -0.74]	1.37 [1.30, 1.44]	0.58 [0.49, 0.67]
			**p<0.001**	**p<0.001**		
***Half-life (days)***		*0*.*96* *[0*.*91*, *1*.*0]*	*NA*	*NA*	*0*.*51* *[0*.*48*, *0*.*53]*	*1*.*2* *[1*.*0*, *1*.*4]*
**Second phase decay rate (days** ^**-1**^ **)**	*b*_*2*_	0.064 [0.046, 0.082]	--	-0.031 [-0.050, -0.012]	0.064 [0.046, 0.082]	0.032 [0.006, 0.059]
			p>0.05	**p = 0.001**		
***Half-life (days)***		*11* *[9*, *15]*	*NA*	*NA*	*11* *[9*, *15]*	*21* *[12*, *110]*

### NK and T cell activation by N-803 in infant macaques

**Fig 4:**
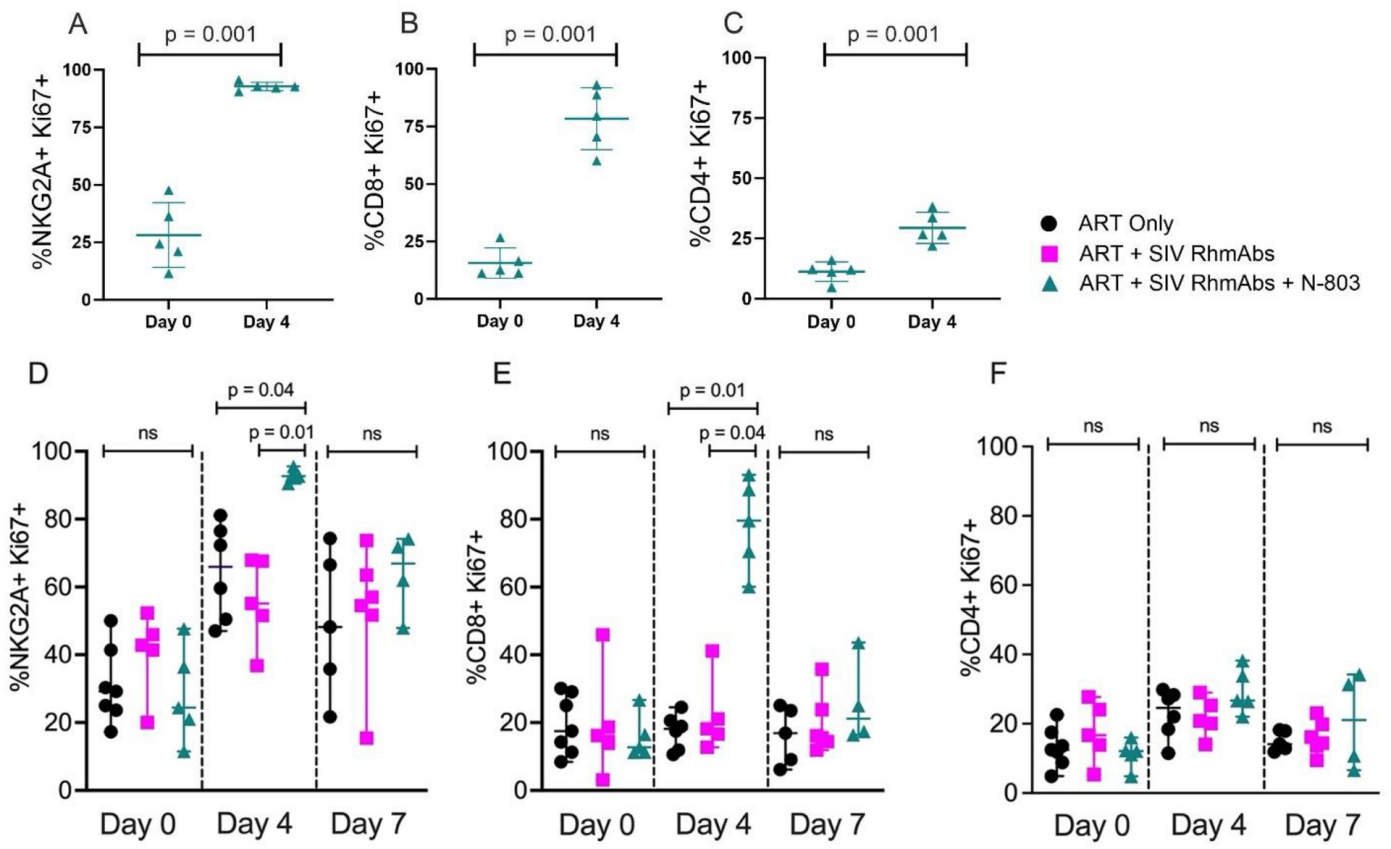
Increased frequency of NK and T cells expressing Ki67 in the ART + SIV RhmAbs + N-803 group. Frequency of NK cells (**A**), CD8+ T cells (**B**), and CD4+ T cells (**C**) expressing intracellular Ki67 measured by whole blood flow cytometry. Frequencies were compared at baseline (day 0) and day 4 after receipt of ART + SIVRhmAbs + N-803. Individual data points are plotted with mean and SD shown. Statistical analysis was performed using a paired Student’s t-test. (**D-F**) Frequency of peripheral blood NK cells (**D**), CD8+ T cells (**E**), and CD4+ T cells (**F**) expressing intracellular Ki67 were compared across groups in the first week after ART +/- SIV RhmAbs +/- N-803 administration. Individual data points are plotted with mean and SD shown. Statistical analysis was performed using the non-parametric Kruskal-Wallis test with multiple comparisons to assess for differences across groups. ns = not significant; p > 0.05.

The impact of N-803 treatment on T and NK cells was evaluated by whole blood flow cytometry. At day 4 after treatment with N-803, the frequency of Ki67+ cells was significantly elevated over baseline in NK, CD8+, and CD4+ T cells (Fig 4A–4C). The ART only group also displayed an increase in Ki67+ NK cells from day 0 to day 4 of ART, but higher expression was found in N-803-treated animals versus the ART only and ART + SIV RhmAbs groups at day 4 (p = 0.04 and p = 0.01, respectively; Fig 4D). The same was found for CD8+ T cells (p = 0.01 for ART + SIV RhmAbs + N-803 versus ART only and p = 0.04 for ART + SIV RhmAbs + N-803 versus ART + SIV RhmAbs; Fig 4E). The activation of T cells was transient, as the frequency of Ki67+ cells declined to baseline levels by day 7 post N-803 treatment; the level of Ki67+ NK cells also declined between day 4 and 7 but remained elevated over baseline. Group differences in Ki67+CD4+ T cell levels were not observed at days 0, 4, or 7. The single dose of N-803 did not result in increases in CCR5 or and PD-1 expression on these cell subsets at either day 4 or 7 (S1 Fig).

### Viral reservoir decay on ART

**Fig 5:**
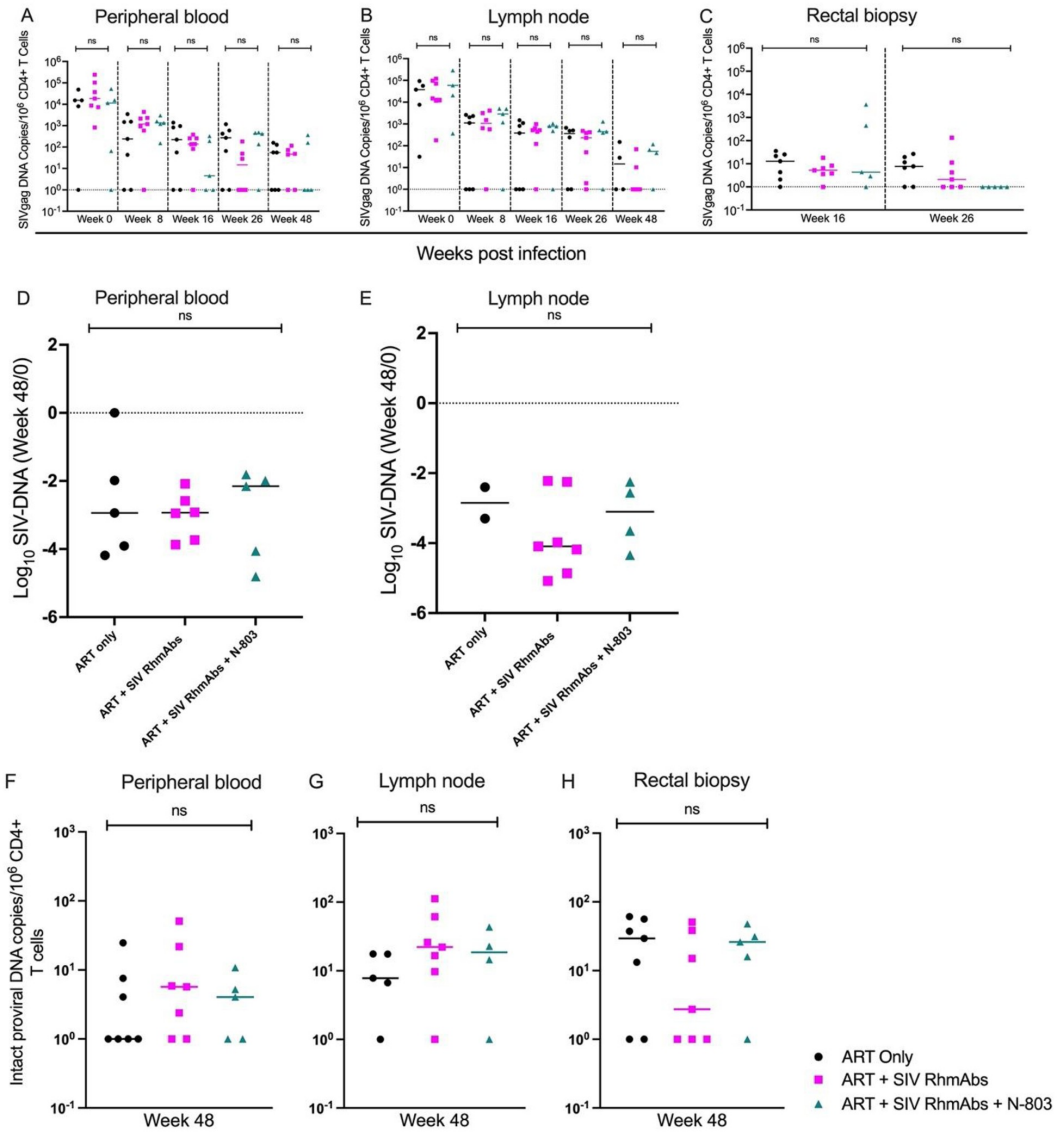
SIV reservoir decay did not differ across groups. SIVgag DNA was quantified in sorted CD4+ T cells from peripheral blood (**A**), lymph nodes (**B**) and rectal biopsies (**C**) starting from week 0 (start of ART) to week 48 of ART. The fold change from week 0 to week 48 was calculated for SIVgag DNA in CD4+ T cells from peripheral blood (**D**) and lymph nodes (**E**). Intact proviral SIV DNA was quantified in sorted CD4+ T cells from peripheral blood (**F**), lymph nodes (**G**), and rectal biopsies (**H**) at week 48 of ART. Individual data points are plotted with horizontal lines drawn at the median. Statistical analysis was performed using the nonparametric Kruskal-Wallis test. Missing data points on graphs are the result of insufficient cells for the respective timepoint or missing paired data in the case of E, ART only group. ns = not significant; p > 0.05.

To evaluate the impact of SIV RhmAbs +/- N-803 at ART initiation on reservoir establishment, cell-associated SIV DNA was tracked longitudinally from pre-ART to week 48 in CD4+ T cells isolated from peripheral blood and lymph nodes. The level of SIVgag DNA in infected CD4+ T cells from peripheral blood similarly declined in all groups, from a peak of ~10^4^ SIV DNA copies per 10^6^ CD4+ T cells pre-ART to ~10^1^ SIV DNA copies per 10^6^ CD4+ T cells at week 48 (Fig 5A). For lymph nodes, SIV DNA in CD4+ T cells declined between week 0 to 8 of ART, stabilized between weeks 8–26, and then trended down at week 48 (Fig 5B). Rectal biopsies were obtained at weeks 16 and 26 and differences between groups were not found in any tissue nor at any time point (Fig 5A–5C), although we note that SIV DNA in rectal CD4+ T cells from the ART + SIV RhmAbs + N-803 group was below the limit of detection at week 26. The fold decline in SIV DNA in peripheral blood and lymph node CD4+ T cells from week 0 to week 48 was similar across groups (Fig 5D and 5E). To further assess the potential effect of the SIV RhmAbs and N-803 on reservoir size, we measured intact SIV proviruses in blood, lymph node, and rectal CD4+ T cells at week 48 following ART initiation. These data, shown in Fig 5F–5H, did not reveal a significant effect of the experimental treatments compared to ART alone.

### SIV rebound kinetics after ART interruption

**Fig 6:**
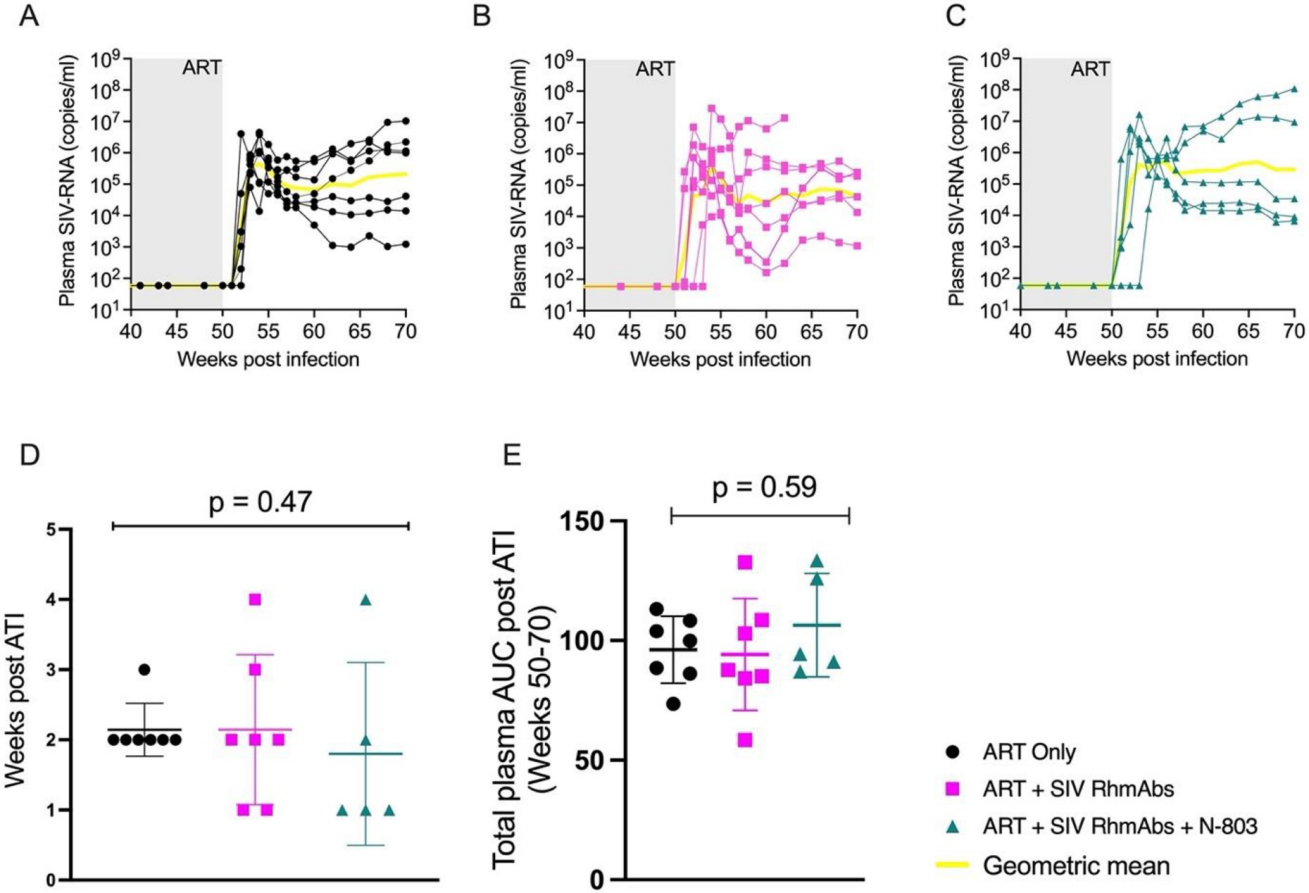
Time to viral rebound and rebound AUC after ART interruption did not differ across groups. (**A-C**), SIV RNA copies in plasma were tracked longitudinally following interruption of ART until euthanasia. (**D**), Time to viral rebound, defined as the first week SIV RNA was >60 copies/ml plasma, was compared across groups. Individual data points are plotted with horizontal line drawn at the mean with SD. Statistical analysis was performed using the nonparametric Kruskal-Wallis test. (**E**), Total area under the curve of rebound plasma viral loads in log for each group was compared. Individual data points are plotted with mean and SD shown. Statistical analysis was performed using the nonparametric Kruskal-Wallis test.

Finally, we evaluated whether the administration of SIV RhmAbs +/- N-803 at the time of ART initiation would have an impact on either the time of viral rebound or rebound set point after ART interruption. ART was thus stopped at 50 weeks post infection and SIV RNA in plasma was measured weekly up to week 57, then bi-weekly until week 70. Most RMs rebounded within 2 weeks of ART interruption and, not unexpectedly given the similar intact SIV DNA levels. There were no differences seen in time to viral rebound across groups (Fig 6A–6D). Viral set points and total area under the curve of viral rebound were also similar (Fig 6E).

## Discussion

Strategies to lower reservoir seeding at the time of ART initiation in infants could reduce viral burden and facilitate HIV cure. Immunotherapies such as bNAbs are an attractive adjunct to ART given that they can be less frequently dosed and have already been demonstrated to be safe [26–28]. bNAbs can facilitate viral suppression due to their Fab mediated neutralizing activity but these and other virus-specific non-neutralizing antibodies also demonstrate Fc mediated effector functions [12,29–31]. Antibody-mediated clearance of HIV-infected cells depends on maintained Env expression as well as immune cell activity and thus we hypothesized that pairing bNAbs with an immunostimulatory agent may be needed to reveal the full activity of passive bNAb immunization. We tested this “surge and purge” strategy here in infant rhesus macaques infected with SIV and treated with early ART. The SIV RhmAbs we used synergized with ART to accelerate first phase decay of viremia; however, the addition of N-803 had an opposing effect, leading to prolongation of the first and second phases of viral decay on ART. We also did not find a reduction in reservoir size or time to viral rebound in the treatment groups compared to macaques that received ART alone.

The idea to disrupt reservoir establishment at the start of ART stems partially from the concept that the reservoir is stabilized at the time of ART initiation [32–34] although we note that there is also a large body of evidence for formation of the latent reservoir within days of infection [35–37]. Two clinical studies have used bNAbs with ART with the goal of reducing reservoir seeding [7,27] and both reported lack of an observed effect on viral DNA-containing cells, like our findings. One of these studies found a higher frequency of post-treatment control following ART interruption in bNAb-treated participants with virus that was sensitive to the bNAb (3BNC117) [7]. There was also an observed enhancement of antiviral CD8+ T cell responses [38], that has been called the ‘vaccinal effect’. As we did not find evidence of increased post-ART viral control in the SIV RhmAb-treated infant macaques, it is unlikely that a vaccinal effect occurred. One potential reason is that in our study ART was started within 1–2 weeks of SIV infection, meaning that the infants may not have had sufficient time to develop the antiviral CD8+ T cell response that would be boosted by antibody administration. In contrast, the participants who experienced post-ART viral control after receiving 3BNC117 at ART initiation were approximately equally distributed amongst those with “recent” (< 6 months) and “long-term” (> 6 months) infection and all had detectable HIV-specific CD8+ T cells [7].

There are several additional differences between our approach and that of prior studies using bNAbs at ART initiation that merit consideration. Firstly, the virus, species, and age of participants all differed. Secondly, we used N-803 for its known ability to activate CD8+ T and NK cells during HIV/SIV infections [15,18–20] while Khaitan et al did not give anything other than the bNAb VRC01 with ART [27] and Gunst and colleagues administered the HDAC inhibitor Romidepsin, a weak latency reversal agent [7]. N-803 has also been described to have latency reversal properties [39,40] although conflicting results have been published from studies of SIV-infected macaques [18–20,41]. Thirdly, 3BNC117 +/- Romidepsin were given starting after one week of ART [7] whereas we elected to treat with SIV RhmAbs +/- N-803 coincident with ART initiation in the hopes of achieving RhmAb binding to the greatest number of infected cells. With all these differences, it is perhaps striking that accelerated decay of viremia in bNAb-treated groups without a clear reduction in the intact reservoir was found in both our study and in [7].

The plasma viral load decline caused by ART is attributed to the half-lives of free virus and productively infected cells since ART halts new rounds of infection. Modeling studies have attributed short-lived activated CD4+ T cells to the first phase, while longer-lived resting memory CD4+ T cells and myeloid cells may contribute to the second and third phases of viral decay on ART [42–44]. In this context, the effect of antibodies and N-803 might be expected to be synergistic in promoting clearance of circulating virions and productively infected cells thus hastening suppression of viremia. In fact, we observed an antagonistic effect, with SIV RhmAbs accelerating first phase viral decay while the addition of N-803 led to slowing of both first and second phase decay compared to macaques treated with ART alone. Our interpretation of this result along with the similar reservoir size and rebound kinetics across groups is that the SIV RhmAb cocktail used did not sufficiently target the cells that contribute to the persistent, rebound competent reservoir and that the pleiotropic immune stimulation caused by N-803 had variable consequences. In other words, the faster decay of viremia observed in the ART + SIV RhmAbs group, likely due to a combination of antiviral effect and clearance of productively infected cells through Fc effector functions, was not accompanied by a decrease in latently infected cells. N-803 had the opposite effect on decay of viremia, meaning that the lifespan of productively infected cells and presence of viruses in circulation was longer. While the latter could be due to latency reversal, the former was likely due to the rapid increase in proliferating CD4+ T cells (as measured by the frequency of Ki67+ cells, Fig 4C). In terms of impact on reservoir, N-803-mediated reversal of latency would be expected to result in a reduction of latently-infected cells through increased viral antigen expression followed by increased RhmAb binding and immune clearance, but CD4+ T cell proliferation could result in expansion of the latently-infected cell pool.

The main limitation of this study is the small sample size, particularly of the group that received the single dose of N-803. The unanticipated adverse events, including death of two infant macaques, within 1–2 days of the first dose of ART (TFV+FTC+DTG) combined with the SIV RhmAbs and N-803 in the setting of acute SIV_mac251_ infection, preclude definitive conclusions. In terms of clinical safety, studies thus far have reported manageable side effects after N-803 infusions in individuals with ART-suppressed HIV infection [39]. There is an ongoing trial of N-803 in adults with acute HIV infection in Thailand (NCT04505501) and there have been no safety signals to date. We note that this was the first study of N-803 in a pediatric setting and expect that dosing will need to be optimized should further studies be pursued. Repeated administrations of the dose used here (0.1 mg/kg) have been previously tolerated in multiple prior adult nonhuman primate studies [18–20,41], but we note that clinical trials in human adults with HIV have tested ~15 times lower doses [39].

The results reported here support the concept that the rebound-competent viral reservoir is formed within days after infection and that targeting productively infected cells for clearance during initial viral suppression on ART does not limit reservoir establishment. There are a few obvious ways in which the approach pursued here could be refined based on these data. A bona fide latency reversal agent at the appropriate dose and frequency could be used at the start of ART to more efficiently promote viral antigen expression from the latently infected cells without causing CD4+ T cell proliferation. One such example could be a mimetic of the second mitochondrial activator of caspases (SMAC) that we have shown induces systemic virus reactivation on ART in absence of global CD4+ T cell activation by targeting the non-canonical NF-kB pathway [45]. Newer antiretroviral drugs that enhance bNAb recognition of viral Env and thus antibody binding to and clearance of infected cells could also be tested [46]. To enhance the likelihood that virus-specific CD8+ T cells may be boosted by the vaccinal effect of bNAbs, ART could be started slightly later in infection. There may be a critical window between the appearance of antiviral T cells and the onset of immune exhaustion that should be targeted. Additional immunotherapeutics such as TLR agonists could also be utilized to strengthen immune responses.

In summary, we found that giving SIV Env RhmAbs +/- a single dose of N-803 at ART initiation modulates viral decay rates without disrupting viral reservoir establishment in SIV-infected infant macaques. Future work should focus on novel approaches to safely target latently infected cells to reduce reservoir formation and stimulate antiviral immunity during initial treatment with ART in infants.

## Methods

### Ethics statement

All animal procedures were performed in accordance with the NIH’s Guide for the Care and Use of Laboratory Animals, 8th edition and guidelines established by the Emory University Institutional Animal Care and Use Committee (IACUC) with approval protocol number PROTO202100117.

### Experimental design, infection, and treatments

Twenty-one infant rhesus macaques of Indian-origin negative for the Mamu B*08 and B*17 alleles were selected for this study from the Emory National Primate Research Center (ENPRC) field station to group housed dams (mothers). The ENPRC is accredited by both the U.S. Department of Agriculture (USDA) and by AAALAC international. All animal procedures were performed in accordance with the NIH’s Guide for the Care and Use of Laboratory Animals, 8^th^ edition and guidelines established by the Emory University Institutional Animal Care and Use Committee (IACUC). Infant macaques were removed from the dams at approximately 2 weeks of age and transferred to an indoor nursery, where they were pair housed with either full contact or protected contact for the duration of the study. The infants were fed in accordance with the ENPRC standard operating procedures for nonhuman primate (NHP) feeding and animal weights were monitored throughout the course of the study. Infant RMs were orally infected at 4 weeks of age with two consecutive doses of 10^5^ TCID_50_ of SIV_mac251_ provided by Dr. Koen Van Rompay of the University of California National Primate Research Center. Some infants required multiple weekly 2-dose challenges before successful viral acquisition (S1 Table). Following infection, infants were sorted into three arms distinguished by their treatment plan. Group 1 received ART only and served as the control group, Group 2 received ART + SIV RhmAbs, while Group 3 received ART + SIV RhmAbs + N-803. Only one dose of N-803 was administered. All treatments began concurrently. All twenty-one infants were treated with a 3-drug ART regimen implemented 1–2 weeks post SIV_mac251_ infection. ART formulation was performed in the lab involving two reverse transcriptase inhibitors and an integrase inhibitor: 5.1 mg/kg tenofovir disoproxil fumarate (TDF), 40 mg/kg emtricitabine (FTC), and 2.5 mg/kg dolutegravir (DTG). This formulation was administered once daily subcutaneously (s.c.) at 1 mg/kg. A cocktail of four SIV Env-targeting RhmAbs (ITS09.01-LS, ITS102.01-LS, ITS103.01-LS, and ITS113.01-LS) were administered at 20 mg/kg each (total of 80 mg/kg) s.c. Group 3 animals also received N-803 s.c at 0.1 mg/kg in addition to the RhmAbs. Fig 1A summarizes the study design.

### Safety assessments

Clinical appearance, weight, complete blood counts, and serum chemistries were monitored following SIV infection and experimental treatments. Infant macaques treated with ART + SIV RhmAbs + N-803 experienced a range of mild to severe adverse effects following administration of a single intervention of the three combined compounds containing N-803, SIV RhmAbs and ART, followed by ongoing ART (S2 Table). In two cases, the severity of the symptoms necessitated euthanasia. A thorough review by study staff, veterinarians, IACUC, and outside experts could not definitively distinguish the intervention that was responsible for the adverse effects and the combination was limited to a single dose.

### Sample collection and processing

Blood samples were collected longitudinally at specific time points over the course of the study and was used to perform routine clinical and laboratory analyses such as blood counts, serum chemistries and immunostaining. Peripheral blood mononuclear cells (PBMCs) were then isolated from the blood by density gradient centrifugation. Lymph nodes (LNs) were collected at specific timepoints throughout the study and mechanically disrupted to remove connective tissue over a 70 μm cell strainer into a single-cell suspension, which was subsequently counted for staining and storage at -80°C. Rectal biopsies were also collected at strategic timepoints of the study, and enzymatically digested with collagenase and DNAse 1 for 2 hours at 37°C, and then also passed through a 70 μm cell strainer into a single-cell suspension, which was subsequently counted for staining and storage at -80°C [24,25,47]. Animals were euthanized at the end of the study, tissue samples collected and cryopreserved for future downstream applications.

### RhmAb concentrations

Concentration of the RhmAbs in plasma was determined by a slightly modified form of Enzyme-linked immunosorbent assay (ELISA) using anti-RhmAb (mouse) mAbs immobilized for capture and anti-rhesus-horseradish peroxidase (HRP) for detection previously described [14]. All ELISAs used total volumes of 100 μl/well (except 250 μL/well for blocking), with coating buffer overnight in PBS at 4°C. All other incubations were performed at 37°C for 1 hour. Binding of the ITS mAb was detected using an anti-rhesus IgG Fc-HRP (Southern Biotech; clone: SB108a). Plates were read on the BioTek 800TS microplate reader.

### Plasma viral load measurement

SIV_mac251_ plasma viral load quantification was performed at the Translational Virology Core Laboratory of the Emory Center for AIDS Research (CFAR) each time blood was collected, using a standard quantitative polymerase chain reaction (PCR) assay with a sensitivity of 60 copies per mL of plasma as its limit of detection as previously described [24,25,47].

### Immunophenotyping by flow cytometry

Flow cytometric analyses was performed on 300 μL of whole blood (WB), and 300 μL LN cell suspension containing about 2 million mononuclear cells. Staining was achieved by labelling WB or LN cells to optimal concentration of fluorescently conjugated monoclonal antibodies. Monoclonal antibodies used in this study include CCR5-Brilliant Violet (BV650), CD3-allophycocyanin (APC)-Cy7 (clone SP34-2), CXCR5-PETR, CD20-Brilliant ultra violet (BUV395), CD56-BUV496, CD16- PECy5, CD95-Brilliant Violet 605 (BV605), Ki67-BD Horizon Red (R718), CCR7-fluorescein isothiocyanate (FITC) (clone 150503), CCR5-Brilliant (Violet BV650), CD62L- R-phycoerythrin (PE), CD45-RA-PE-Cy7 (clone L45) from BD Biosciences; CD8-BV711 (clone RPA-T8), CD4-Brilliant Violet (BV750), and PD-1-BV421 (clone EH12.2H7) from BioLegend; NKG2A-APC, CD28-PE-Cy5.5 from Beckman-Coulter. Flow cytometric acquisition and analysis of samples was performed on at least 1,000,000 events on an AURORA flow cytometer driven by the Cytek SpectroFlo software package (Cytek). Analyses of the acquired data were performed using FlowJo version 10.0.4 software (TreeStar).

### CD4+ T cell enrichment by negative selection

Mononuclear cells were counted and placed in 15 mL or 50 mL conical tubes depending on the sample volume. Negative selection using CD4+ T cell isolation kits for nonhuman primates (Miltenyi Biotec) was performed according to the Manufacturer’s recommendations. Recovered CD4+ T cells were then pelleted and prepared for subsequent nucleic acid extraction. CD4+ T cell enrichment and nucleic acid extraction were performed on mononuclear cells recovered from rectal biopsies immediately to improve yield.

### Viral reservoir quantification

Cell-associated SIV_mac251_ DNA was also performed at the Translational Virology Core Laboratory of the Emory Center for AIDS Research (CFAR). Each time blood was collected, total SIV DNA levels were measured in total CD4+ T cells negatively selected from isolated (PBMCs), lymph node mononuclear cells (LNMCs) and rectal biopsy mononuclear cells (RBMCs) ranging from 1,000,000–3,000,000 cells lysed in Buffer RLT Plus (Qiagen) plus 2-mercaptoethanol. Both DNA and RNA were extracted using the Allprep DNA/RNA minikit (Qiagen). Quantification of SIV_mac251_ gag DNA was performed on the extracted DNA by quantitative PCR using the 5’ nuclease (TaqMan) assay with an ABI7500 system (PerkinElmer Life Sciences) [47]. An SIVmac251 adapted IPDA was also performed by Accelevir Diagnostics to quantify intact proviral DNA at specific timepoints using methods previously described [48–50].

### Statistical analysis

Graphs were generated with Prism v10 (GraphPad). Parametric and nonparametric methods including the Student t, Kruskal-Wallis and Mann-Whitney tests were used to evaluate differences within a group and across treated and untreated groups and p values less than 0.05 were considered significant. A two-phase non-linear regression analysis was performed to determine the pharmacokinetic parameters for SIV RhmAbs concentrations in GraphPad PRISM.

### Viral decay modeling

The kinetics of viral decay following the initiation of ART were quantified. Following previous work [51], we assumed that viral dynamics were described by a simple mechanistic model in which two subpopulations of infected cells—shorter-lived and longer-lived–may release virus before dying, and that ART completely blocks new infection of target cells. In this model, the shorter-lived infected cells—which contribute to the “first phase” of viral decay—are believed to represent activated, terminally differentiated CD4+ T cells. The identity of the cells contributing to the “second phase decay” is still unsettled but is separate from cells comprising the latent reservoir which decays at an even longer timescale and typically produces levels of viremia below the detection limit of assays used. With this model, the decay of the viral load over time (V(t)) follows a biphasic exponential function:

$$V\left(t\right)=V_{0}((1–A)e^{{-b}_{1}t}+Ae^{{-b}_{2}t})$$

where *V*_*0*_ is the pre-treatment viral load, *A* is the proportion of infected cells in the long-lived vs short-lived state, *b*_*1*_ is the first phase decay rate, and *b*_*2*_ is the second phase decay rate. We used a log10 transformation of the biphasic exponential model to better constrain the scale of the parameters, and instead estimate *v*_*0*_ = log10(*V*_*0*_) and *a* = log10(*A*) along with *b*_*1*_ and *b*_*2*_.

We used a nonlinear mixed-effects statistical modeling framework to examine the effects of adding RhmAbs or RhmAbs + N-803 to ART, as compared to ART-only, on the viral decay dynamics. This framework assumes that individual viral decay follow the same model but with inter-individual variation in the model parameters. We model this individual variation by assuming that for individual *j*, the model parameters *p*_*j*_ (where *p* is one of *v*_*0*_, *a*, *b*_*1*_ or *b*_*2*_) can be described by:

$${\text{p}}_{\text{j}}={\text{p}}_{\text{pop}}+\beta_{\text{RhmAbs},\text{p}}\;{\text{i}}_{\text{RhmAbs},\text{j}}+\beta_{\text{N}803,\text{p}}\;{\text{i}}_{\text{N}803,\text{j}}+\varepsilon_{\text{p},\text{j}}$$

where *p*_*pop*_ is the population mean of the parameter in the absence of RhmAbs or N-803 treatment (the “fixed effects”), β_x,p_, corresponds to the shift in the parameter *p* for individuals receiving treatment X (either RhmAbs or N-803, the “treatment effects”), the *i*_*j*_ are binary variables (value 0 or 1) indicating which treatments were received by individual *j*, and ε_p, j_ are individual-level shifts in parameters from the population-mean, drawn from a normal distribution with mean zero and standard deviation *σ*_*p*_ (“random effects”).

We estimated model parameters by jointly fitting the model to the viral load data of all individual animals. Fitting was done using Monolix 2023R1 (Lixoft) using the Stochastic Approximation Expectation-Maximization algorithm (SAEM). We used the observed log10 of plasma viral loads (RNA copies/mL) sampled from the time of ART until 336 days after in model fitting and assumed a constant error model for the observed values. Viral load values below the limit of detection were recorded and fit as “censored” at 60 copies per mL. We used a combined forward-backward selection procedure to a) identify the parameters for which there was evidence of significant variation between individuals and thus the need for inclusion of random effects, and b) identify the parameters for which there was evidence of significant variation by treatment received.

To identify significant random effects, we started with random effects on all four model parameters, and then removed the random effects one at a time to verify that each parameter’s random effects are improving the model. We used the BIC as target criterion, with a threshold of improvement of ΔBIC>2. Removing random effects on the parameters *v*_*0*_, *a*, and *b*_*1*_ leads to increases in ΔBIC>2. However, there was not strong evidence supporting the inclusion of random effects on *b*_*2*_ (ΔBIC<2). We therefore included random effects on *v*_*0*_, *a*, and *b*_*1*_, but removed the random effects on *b*_*2*_ for the remainder of the analysis.

To determine which of the four model parameters are significantly impacted by RhmAbs or N-803 treatment, we used the Stepwise Covariate Modeling (SCM) method, starting from a model with no treatment effects included. The forward portion of this stepwise method examines how including each parameter-covariate relationship affects the model. The models are compared using a likelihood ratio test, and the parameter-covariate relationship resulting in the most improvement in log-likelihood is added to the model in the next step. This stepwise addition of parameter-covariate effects continues until no improvements are observed. In the backward portion of the SCM, for each parameter-covariate effect that was added in the forward section, the model performance with and without the effect is compared using a likelihood ratio test, and any parameter-covariate effects that failed to improve the model performance were discarded. We used the default threshold of 0.05 in the forward phase, and of 0.01 in the backward phase. This procedure identified the optimal model as one in which both RhmAbs and N-803 have significant impacts on the first phase decay rate (*b*_*1*_) and N-803 has a significant impact on the second phase decay rate (*b*_*2*_).

We then ran 100 independent realizations of the parameter estimation, each with a minimum of 200 iterations in the exploratory phase and a minimum of 100 iterations in the smoothing phase. This was implemented using the Rsmlx package in R. We selected the resulting model with the lowest log-likelihood among these 100 realizations. Confidence intervals for the population parameters and treatment effects were estimated from the standard errors derived from the Fisher Information Matrix. Code to reproduce our results is publicly available on Github: https://github.com/annehebert/ViralDecayFits.

## Supporting information

S1 Fig CCR5 and PD-1 Frequencies. (**A**) Frequencies of NK cells, CD8+ T cells, and CD4+ T cells expressing CCR5 measured by whole blood flow cytometry were compared at baseline (day 0) and day 4 or day 7 after receipt of ART + SIV RhmAbs + N-803. Individual data points are plotted with mean and SD shown. (**B**) Frequencies of peripheral blood NK cells, CD8+ T cells, and CD4+ T cells expressing CCR5 were compared across groups in the first week after ART +/- SIV RhmAbs +/- N-803 administration. Individual data points are plotted with mean and SD shown. (**C**) Frequencies of NK cells, CD8+ T cells, and CD4+ T cells expressing PD-1 measured by whole blood flow cytometry were compared at baseline (day 0) and day 4 or day 7 after receipt of ART + SIV RhmAbs + N-803. Individual data points are plotted with mean and SD shown. (**D**) Frequencies of peripheral blood NK cells, CD8+ T cells, and CD4+ T cells expressing PD-1 were compared across groups in the first week after ART +/- SIV RhmAbs +/- N-803 administration. Individual data points are plotted with mean and SD shown. For (**A**) and (**C**) statistical analysis was performed using the non-parametric Kruskal-Wallis test. For (**B**) and (**D**) statistical analysis was performed using the non-parametric Kruskal-Wallis test with multiple comparisons to assess for differences across groups.
(TIF)

S1 Table Characteristics of Study Groups. (DOCX)

S2 Table Adverse Events (AEs). (DOCX)

S1 Data Source Data. (XLSX)
