# Supplementary figures and images for: SIV Env RhmAbs + N-803 at ART initiation prolongs viral decay without disrupting reservoir establishment in SIV-infected infant macaques
Source: PLoS Pathog. 2025 Jan 10;21(1):e1012863. doi: 10.1371/journal.ppat.1012863 (PMC11756789; doi:10.1371/journal.ppat.1012863)

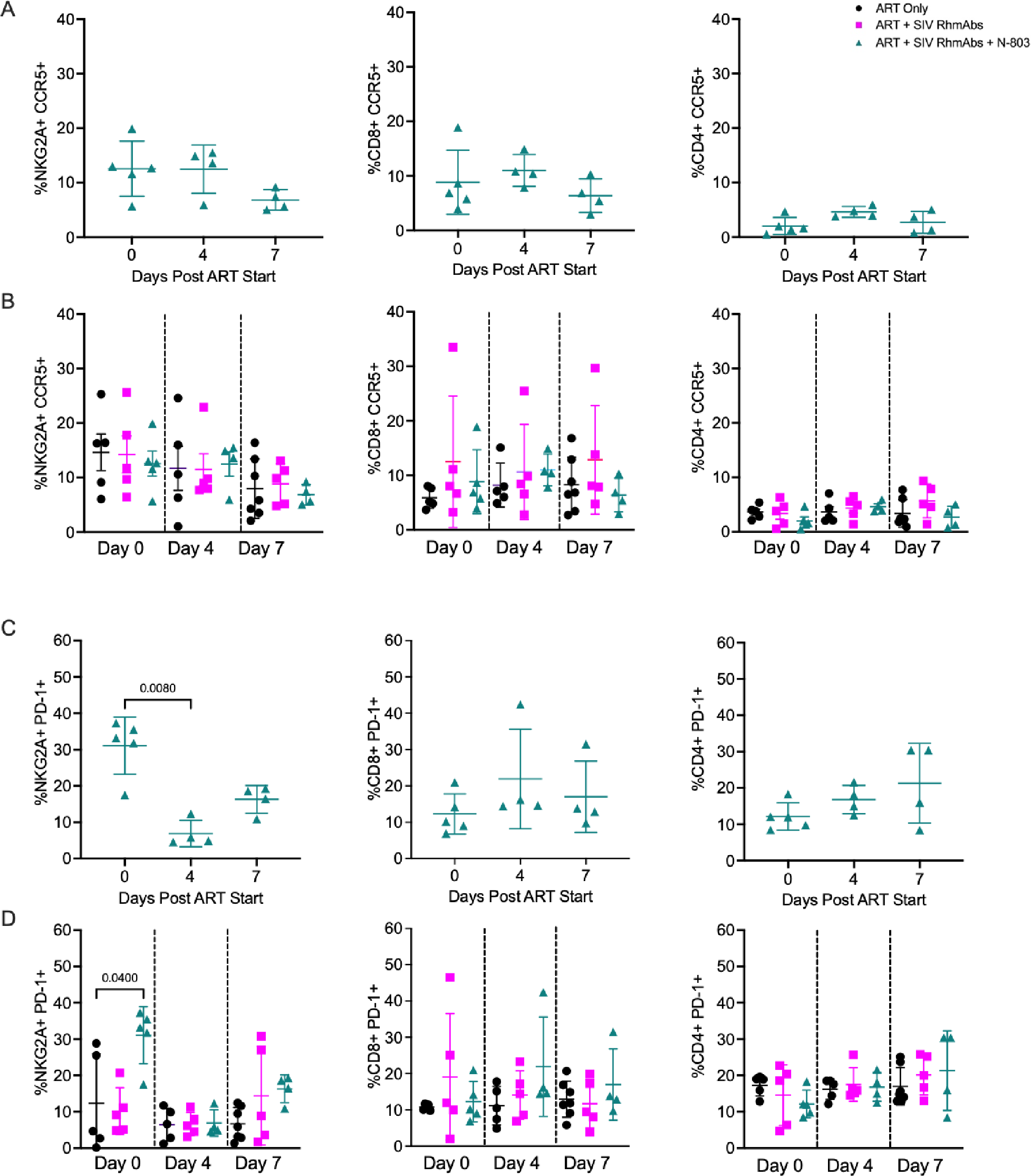

Supplement: S1 Fig — (A) Frequencies of NK cells, CD8+ T cells, and CD4+ T cells expressing CCR5 measured by whole blood flow cytometry were compared at baseline (day 0) and day 4 or day 7 after receipt of ART + SIV RhmAbs + N-803. Individual data points are plotted with mean and SD shown. (B) Frequencies of peripheral blood NK cells, CD8+ T cells, and CD4+ T cells expressing CCR5 were compared across groups in the first week after ART +/- SIV RhmAbs +/- N-803 administration. Individual data points are plotted with mean and SD shown. (C) Frequencies of NK cells, CD8+ T cells, and CD4+ T cells expressing PD-1 measured by whole blood flow cytometry were compared at baseline (day 0) and day 4 or day 7 after receipt of ART + SIV RhmAbs + N-803. Individual data points are plotted with mean and SD shown. (D) Frequencies of peripheral blood NK cells, CD8+ T cells, and CD4+ T cells expressing PD-1 were compared across groups in the first week after ART +/- SIV RhmAbs +/- N-803 administration. Individual data points are plotted with mean and SD shown. For (A) and (C) statistical analysis was performed using the non-parametric Kruskal-Wallis test. For (B) and (D) statistical analysis was performed using the non-parametric Kruskal-Wallis test with multiple comparisons to assess for differences across groups. (TIF) [file ppat.1012863.s001.tif]
