# Supplementary material for: SIV Env RhmAbs + N-803 at ART initiation prolongs viral decay without disrupting reservoir establishment in SIV-infected infant macaques
Source: PLoS Pathog. 2025 Jan 10;21(1):e1012863. doi: 10.1371/journal.ppat.1012863 (PMC11756789; doi:10.1371/journal.ppat.1012863)
Supplement: S1 Table — (DOCX) [file ppat.1012863.s002.docx]

**S1 Table. Characteristics of study groups.**

| **Group** | **Animal I.D.** | **Sex** | **A01 status** | **Age at infection (weeks)** | **Number of oral SIV challenges** | **Peak SIV RNA copies/ml of plasma** |
| --- | --- | --- | --- | --- | --- | --- |
| **ART** | RAn22 | Male | - | 4 | 1 | 1.07E+07 |
|  | RDm22 | Male | - | 4 | 1 | 9.01E+06 |
|  | RGn22 | Male | - | 4 | 1 | 2.57E+07 |
|  | RQl22 | Male | - | 4 | 1 | 1.06E+07 |
|  | RBm22 | Female | + | 6 | 2 | 1.02E+07 |
|  | RWl22 | Female | - | 6 | 2 | 1.87E+07 |
|  | RKr22 | Male | + | 8 | 4 | 1.83E+04 |
| **ART + SIV RhmAbs** | RCt22 | Male | - | 4 | 2 | 5.72E+06 |
|  | RFo22 | Male | + | 4 | 2 | 2.58E+07 |
|  | RIn22 | Female | - | 4 | 2 | 6.25E+08 |
|  | RKn22 | Female | - | 4 | 2 | 5.11E+06 |
|  | RNs22 | Female | - | 4 | 2 | 2.47E+06 |
|  | RYn22 | Female | - | 4 | 2 | 2.17E+06 |
|  | RUm22 | Male | - | 7 | 3 | 7.08E+06 |
| **ART + SIV RhmAbs + N-803** | REp22 | Female | - | 4 | 2 | 4.38E+07 |
|  | RLp22 | Female | - | 4 | 2 | 9.21E+07 |
|  | RNq22 | Male | - | 4 | 2 | 7.46E+07 |
|  | RTo22 | Male | + | 4 | 2 | 3.40E+07 |
|  | RZo22 | Male | + | 4 | 2 | 4.13E+04 |
