# Supplementary material for: SIV Env RhmAbs + N-803 at ART initiation prolongs viral decay without disrupting reservoir establishment in SIV-infected infant macaques
Source: PLoS Pathog. 2025 Jan 10;21(1):e1012863. doi: 10.1371/journal.ppat.1012863 (PMC11756789; doi:10.1371/journal.ppat.1012863)
Supplement: S2 Table — (DOCX) [file ppat.1012863.s003.docx]

**S2 Table. Adverse events (AEs).**

| Animal I.D. | Group | Timeline | Description | Treatments | | Outcome | | |
| --- | --- | --- | --- | --- | --- | --- | --- | --- |
| ROq22 | ART  +SIV RhmAbs  + N-803 | 1 d post | Depression, lethargy (moderate); stabilized for < 24 h; depressed mentation, cyanosis (severe) | Antihistamine, fluids; other emergency care | Euthanized | | |  |
| RBr22 | ART  +SIV RhmAbs  + N-803 | 3 d post | Depression, lethargy (moderate); stabilized for < 24 h; depressed mentation, cyanosis (severe) | Antihistamine, fluids; other emergency care | | Euthanized |  |  |
| REp22 | ART  + SIV RhmAbs  + N-803 | 3 d post | Depression, lethargy (mild) | Antihistamine, fluids | | Recovered | | |
| RLp22 | ART  + SIV RhmAbs  + N-803 | 4 d post | Depression, lethargy (mild) | Antihistamine, fluids | | Recovered | | |
| RZo22 | ART  + SIV RhmAbs  + N-803 | 4 d post | Depression, lethargy (moderate) | Antihistamine, fluids, steroids | | Recovered | | |
| RNq22 | ART  + SIV RhmAbs  + N-803 | No AEs | NA* | NA | | NA | | |
| RTo22 | ART  + SIV RhmAbs  + N-803 | No AEs | NA | NA | | NA | | |

* NA = not applicable
